# Supplementary material for: Structure, dynamics and transferability of the metal-dependent polyhistidine tetramerization motif TetrHis for single-chain Fv antibodies
Source: Commun Chem. 2023 Jul 28;6:160. doi: 10.1038/s42004-023-00962-x (PMC10382482; doi:10.1038/s42004-023-00962-x)
Supplement: Supplementary file 2 — Supplementary Information [file 42004_2023_962_MOESM2_ESM.pdf]

Supplementary Figures and tables

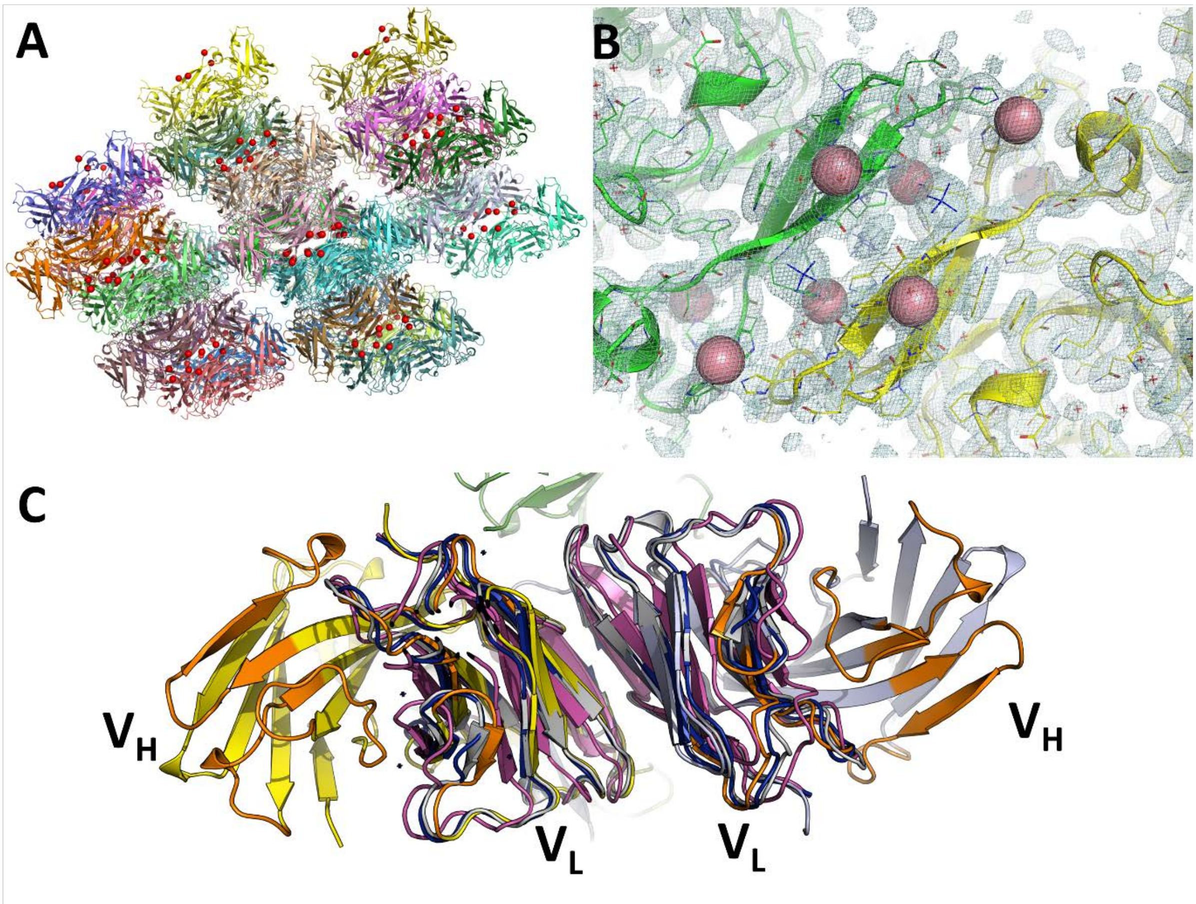

Supplementary Figure 1: Crystal structure of scFv 2A2. A. Crystal packing of the scFv 2A2 structure. The protein chains are shown as cartoon representation and the cobalt items are shown as spheres and colored in red. B. 2Fo-Fc electron density map of the TetrHis motif contoured at 1σ. C. Comparison of the V_L- V_L interface in scFv 2A2 with similar interfaces observed in other scFv (5lwy, magenta and 6j5f, gray) and isolated V_L domain structures (1f6l, dark blue).

PDB code	Number of visible Histag residues	bound metal	Number of metal ions	Number of protein chains with coordinating side chains	linear motif
2fg9	3 x 5	NI	1	3	yes
4bf5	1 x 6	NI	1	2	no
4ehc	1 x 6	ZN	1	1	no
5fib	1 x 6	ZN	1	2	no
5k1s	1 x 5	ZN	1	2	no
6omk	1 x 4	ZN	1	2	no
6vfu	1 x4	NI	1	1	no
6wxa	3 x 4	NI	1	3	yes
7c3a	3 x 5	NI	1	3	yes
2z36	1 x 5	FE	1	2	no
1sf8	1 x 5	NI	2	2	no
4r4x	1 x 8	ZN	2	2	no
2a5v	1 x 6	ZN	2	2	no
3cgm	2 x 5	NI	2	2	yes
4odp	2 x 6	NI	2	2	yes
4zn3	1 x 4	FE	2	2	no
7cpl	1 x 6	NI	2	2	no
2w5e	1 x 6	CD	3	3	no
3dtu	1 x 6, 1 x 4	CD	3	2	no
3nac	1 x 6	ZN	4	2	no
6h1x	2 x 6	CO	4	2	no
6imz	2 x 6	ZN	4	2	no
6jf1	1 x 6	ZN	4	2	no
6r3u	2 x 6	ZN	4	2	no
1vk9	2 x 6	ZN	5	2	no
6fj4	2 x 6	CD	2 x 3	2	no
SCFV 2A2	4 x 6	CO	8	4	yes
1hz5	2 x 6	ZN	9	4	no
3ef7	1 x 5, 2 x 5	ZN	5 + 7	2 + 4	no

Supplementary Table 1: PDB IDs and characteristics of the 27 unique entries identified in the PDB containing at least 4 consecutive visible histidines and any bound zinc, copper, nickel, cobalt, cadmium or iron in interaction with the polyhistidine sequence.

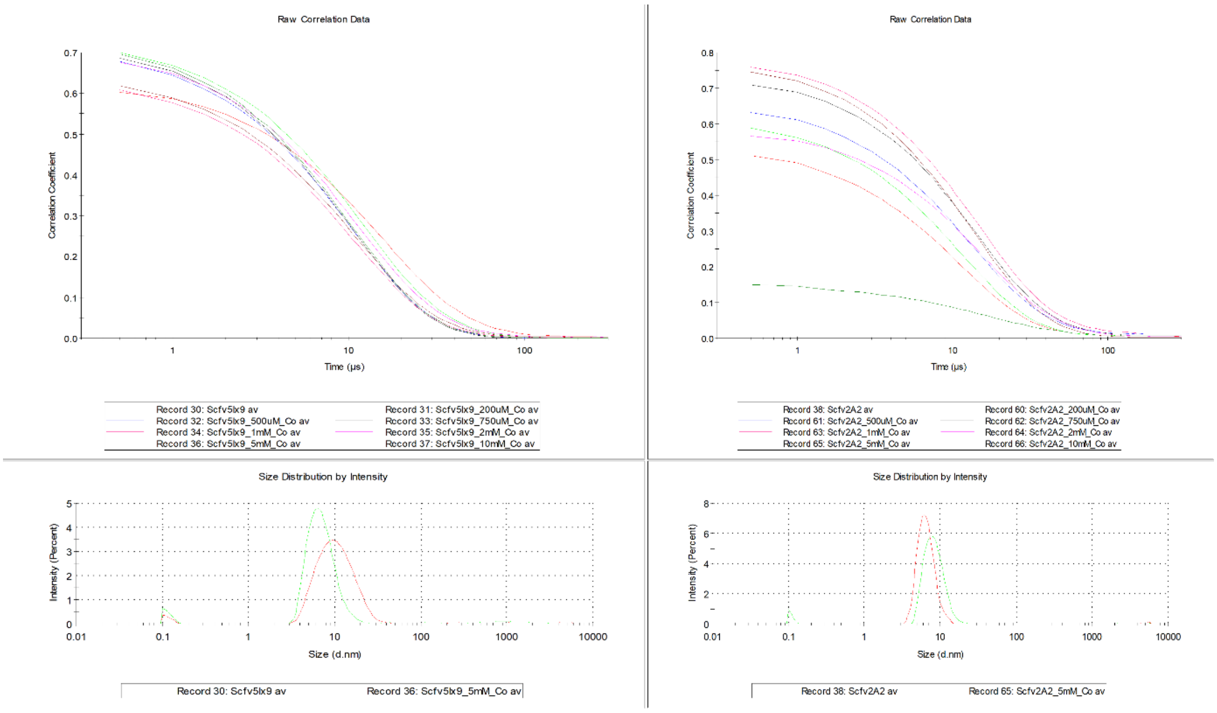

Supplementary Figure 2: Dynamic light scattering data for control anti-ADIPOR scFv (pdb id 5lx9) and scFv 2A2 (left and right panels respectively). The top panels show the raw intensity autocorrelations in regular buffer or in the presence of increasing concentrations of cobalt (II) ions. The bottom panels show a comparison of the relative size distributions in buffer (red) versus 5mM $CoCl_2$ (green).

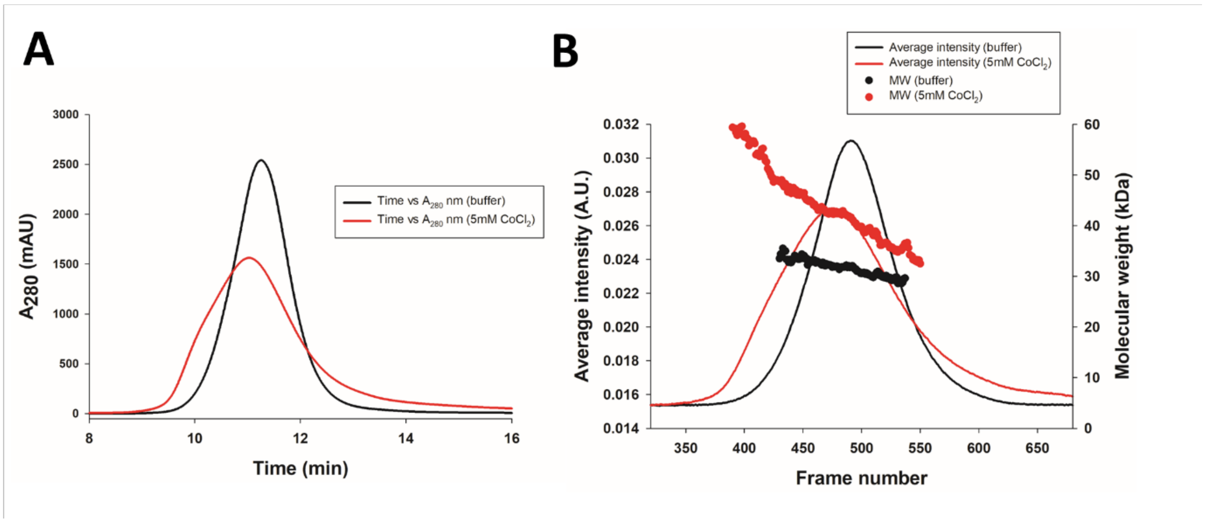

Supplementary Figure 3: Additional SEC-SAXS profiles of scFv 2A2 the presence or absence of 5mM CoCl2 showing the measured absorbance traces at 280 nm (A), and the estimated molecular weight from each data frame (B).

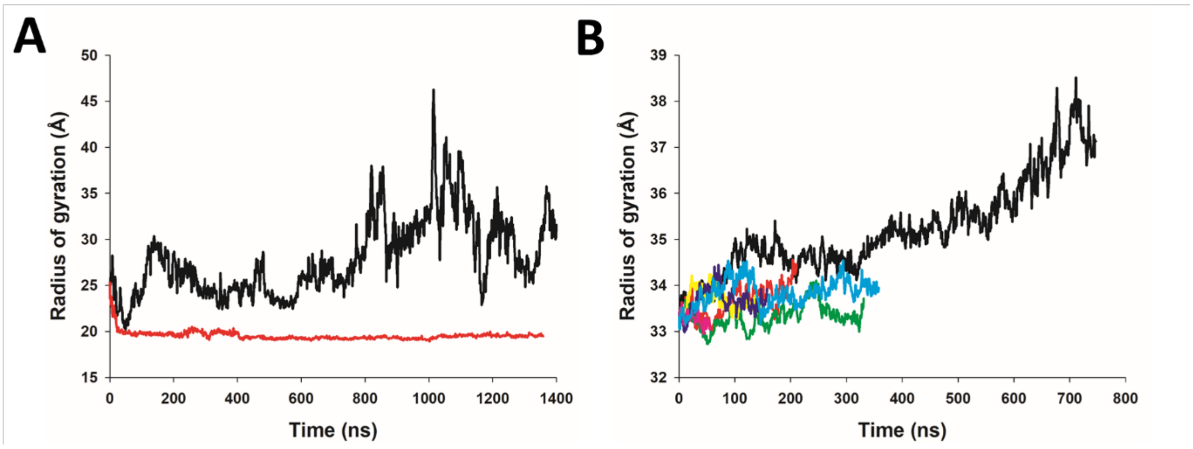

Supplementary Figure 4: radius of gyration versus simulation time for molecular dynamics trajectories of the monomer (A) and the tetramer (B).
