# Supplementary material for: Structure, dynamics and transferability of the metal-dependent polyhistidine tetramerization motif TetrHis for single-chain Fv antibodies
Source: Commun Chem. 2023 Jul 28;6:160. doi: 10.1038/s42004-023-00962-x (PMC10382482; doi:10.1038/s42004-023-00962-x)
Supplement: Supplementary file 3 — Description of Additional Supplementary Files [file 42004_2023_962_MOESM3_ESM.pdf]

Description of Additional Supplementary Files

File name: Supplementary Data 1

Description: Crystal structure of cobalt-bound scFv 2A2 at 2.5 Å resolution.
